# Dr. Edward P. Hay

**Published:** 1901-09

**Authors:** 


### OBITUARY NOTES.

Dr. Edward P. Hay, U. of B., 1899, died at the German Hospital in Buffalo, August 10, 1901.

